# Association Between Mild Thrombocytopenia Prior to Cesarean Section and Postpartum Hemorrhage

**DOI:** 10.3390/jcm14062031

**Published:** 2025-03-17

**Authors:** Kyung-Eun Lee, Eun-Jeong Byeon, Mi-Ju Kwon, Hyun-Sun Ko, Jae-Eun Shin

**Affiliations:** 1 Department of Obstetrics and Gynecology, Bucheon St. Mary’s Hospital, College of Medicine, The Catholic University of Korea, Seoul 06591, Republic of Korea; gangiii@catholic.ac.kr (K.-E.L.); dmsejal@naver.com (E.-J.B.); 2 Department of Obstetrics and Gynecology, Seoul St. Mary’s Hospital, College of Medicine, The Catholic University of Korea, Seoul 06591, Republic of Korea; miju851104@naver.com (M.-J.K.); monkoko@cathlic.ac.kr (H.-S.K.)

**Keywords:** blood transfusion, cesarean section, postpartum hemorrhage, thrombocytopenia

## Abstract

**Objective:** In this study, we aimed to evaluate the impact of mild thrombocytopenia on the incidence of postpartum hemorrhage (PPH) and associated clinical outcomes in a cohort of pregnant women who delivered via cesarean section. **Methods:** Women who underwent cesarean delivery at two tertiary medical centers in Korea between January 2009 and December 2019 were included in this retrospective cohort study. Associations between groups and baseline characteristics were assessed using *t* tests and chi-square tests. Logistic regression was performed to evaluate the increased risk of PPH. All analyses were conducted using R version 4.3.3. **Results:** Of 15,549 women who gave birth, 6487 met the inclusion criteria; 485 (7.5%) were diagnosed with mild thrombocytopenia, whereas 6002 had normal platelet counts. Women with mild thrombocytopenia had a threefold higher risk of PPH (adjusted OR: 3.74; 95% CI: 1.36–10.30) compared to those with normal platelet counts. They were also more likely to require blood transfusions and experience a >4 g/dL drop in hemoglobin level (adjusted OR: 2.28 and 2.99, respectively). In the subgroup analysis, women with immune-related thrombocytopenia had lower platelet counts than other groups from the third trimester to 2 months postpartum. However, primary and secondary PPH outcomes did not differ significantly among the subgroups. **Conclusions:** Mild maternal thrombocytopenia before cesarean section was associated with a higher risk of PPH compared to normal platelet counts; however, the overall prognosis was similar regardless of the underlying cause.

## 1. Introduction

Despite advancements in obstetric care, postpartum hemorrhage (PPH) remains a major contributor to maternal mortality and morbidity worldwide [1,2,3]. The incidence of PPH is increasing globally, including in developed countries [4,5,6,7]. In the United States, the prevalence of PPH rose from 2.9% of deliveries in 2010 to 3.2% in 2014. This alarming trend is particularly concerning, as PPH accounts for approximately 11% of maternal deaths [8]. Identifying risk factors for PPH is essential for implementing targeted improvements in care before delivery, especially during cesarean section, which is more strongly associated with PPH than vaginal delivery [9].

Severe thrombocytopenia, defined as a platelet count <50 × 10^9^/L [10], is a known risk factor for PPH and blood transfusion [11]. Thrombocytopenia complicates approximately 10% of all pregnancies, making it the second most common hematologic disease in pregnant women; however, the association of PPH with mild thrombocytopenia between 100 × 10^9^/L and 149 × 10^9^/L is less clear [12,13,14]. While guidelines from the American College of Obstetricians and Gynecologists indicate that mild thrombocytopenia is usually due to gestational thrombocytopenia (GT), pregnancies with GT are generally not at an increased risk of maternal bleeding complications, and interventions during delivery are not indicated [15]. Nevertheless, studies on the relationship between mild thrombocytopenia and PPH have yielded inconsistent results. Moreover, these studies have mostly focused on the association between GT and PPH, although thrombocytopenia during pregnancy can have various causes. Approximately 75–80% of thrombocytopenia cases are due to GT, 15–20% are due to preeclampsia/HELLP syndrome (hemolysis, elevated liver enzymes, low platelet count), and 1–5% are due to other etiologies, including immune diseases or infection [15,16,17]. As many studies either exclude or do not document other causes of thrombocytopenia [18,19,20,21,22], the effects of mild thrombocytopenia due to various causes on PPH remain unknown.

In this study, we aimed to investigate the effect of mild thrombocytopenia on the incidence of PPH and its associated clinical outcomes. We also performed subgroup analyses to determine the difference in the incidence of PPH according to the cause of mild thrombocytopenia.

## 2. Materials and Methods

### 2.1. Study Design

This retrospective cohort study was conducted at two tertiary centers of the Catholic University of Korea in two different metropolitan cities. Complicated obstetric cases within the region are generally referred to these hospitals, especially for women with hematologic diseases. We used medical record data collected between January 2009 and December 2019, extracted from an electronic database. This study was approved by the Institutional Review Board of each hospital (Approval No. XC20WIDI0103).

### 2.2. Study Population

Women who delivered after 20 weeks of gestation were included in this study. The exclusion criteria were as follows: multifetal pregnancy, vaginal delivery, coagulopathy of unknown origin, missing admission data, and platelet count at admission for delivery of <100 × 10^9^/L. Women with placental abnormalities complicating pregnancy, including placental abruption, placenta previa, or placenta accreta syndrome, were also excluded. The remaining patients were classified based on their preoperative platelet count as normal (at least 150 × 10^9^/L) or mild thrombocytopenia. The risk of PPH during admission for cesarean section was also evaluated. To determine PPH incidence according to the cause of thrombocytopenia, the mild thrombocytopenia group was subdivided into the following three groups: *gestational thrombocytopenia (GT)*, immune-related thrombocytopenia (IT), and pregnancy-induced hypertension (PIH). Women with mild thrombocytopenia during uncomplicated pregnancies were categorized into the GT group, whereas those with thrombocytopenia associated with pregnancy-related complications, such as preeclampsia and HELLP syndrome, were categorized into the PIH group. Women with thrombocytopenia and pre-existing immune-related disorders, such as immune thrombocytopenic purpura (ITP) or systemic lupus erythematosus (SLE), were categorized into the IT group.

### 2.3. Outcomes

PPH was primarily defined using ICD-10 codes, with secondary outcomes including a >4 g/dL drop in postpartum hemoglobin (Hb) from admission to the first postoperative day, as well as the need for an intrauterine device, uterine artery embolization (UAE), hysterectomy, and blood transfusion. Women with excessive blood loss received additional perioperative or postpartum uterotonic agents, including oxytocin, carbetocin, methylergonovine, or misoprostol. If bleeding persisted despite medical treatment, intrauterine device placement, UAE, or hysterectomy was considered. Blood transfusion was considered based on perioperative blood loss, vital signs, and postoperative hemoglobin levels.

### 2.4. Covariates

Pre-gestational and obstetric characteristics, including maternal age, primiparity, gestational age at delivery, and neonatal birth weight, were identified using hospital databases. Pre-gestational maternal body mass index (BMI) was calculated by dividing the pre-pregnancy weight by height squared. Using ICD-10 codes, additional data on underlying diseases in women, including immune-related hematologic diseases and pre-pregnancy hypertension, were also evaluated. Information on pregnancy complications such as PIH, gestational diabetes mellitus, and uterine atony was also obtained using ICD-10 codes. PIH was defined using ICD codes for gestational hypertension, preeclampsia, superimposed preeclampsia, eclampsia, and HELLP syndrome. Data on previous uterine operations, emergency cesarean sections, and blood samples were obtained from electronic medical records. An elective cesarean section was defined as one planned before the day of delivery, whereas an emergency cesarean section was defined as one performed within 24 h of planning due to labor, vaginal bleeding, or other complications affecting the maternal condition. Mean platelet counts were achieved longitudinally at the following time points: pregestational period (within 6 months), each trimester of the antepartum period, at admission for delivery, postoperative days 1 and 3, and postpartum period (within 8 weeks after delivery).

### 2.5. Statistical Analysis

Continuous and categorical variables are expressed as mean ± standard deviation and percentages, respectively. Baseline characteristics and longitudinal blood sample results were compared between women with normal platelet counts and those with mild thrombocytopenia using the *t* test for continuous variables and the chi-square test for categorical variables. Logistic regression analysis was performed to explore the relationship between mild preoperative thrombocytopenia and PPH in women who underwent cesarean delivery. For multivariate analysis, we adjusted for factors such as maternal age, gestational age at delivery, maternal BMI, and previous abdominal surgery, including cesarean section. Additional analysis was performed to assess the persistence of the relationship between PPH and mild thrombocytopenia in the GT subgroup after excluding the PIH and IT subgroups. In the subgroup analysis, the primary and secondary outcomes of PPH were compared among the three subgroups. Statistical significance was set at a *p*-value <0.05. All analyses were performed using R version 4.3.3 (R Foundation for Statistical Computing, Vienna, Austria).

## 3. Results

**Figure 1:**
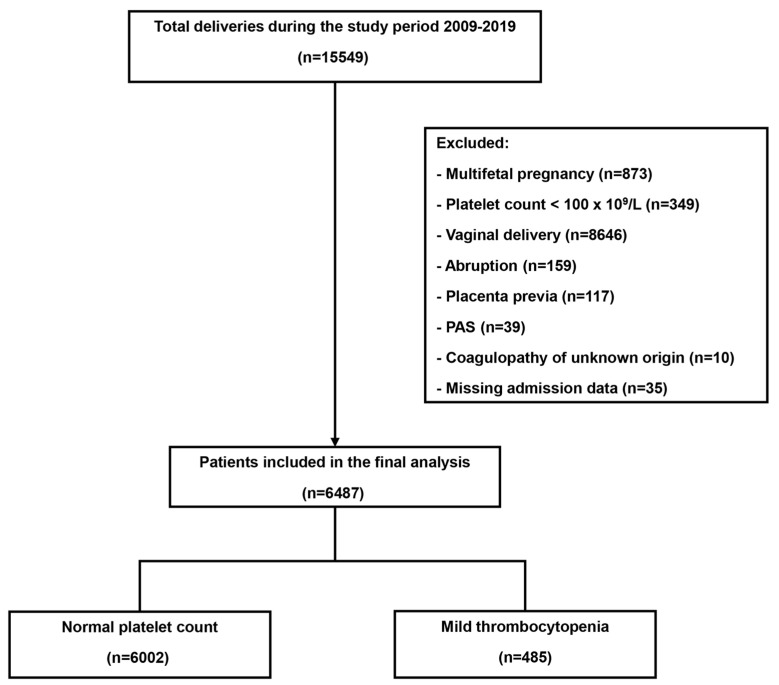
Flow diagram of the study population.

Of 15,549 mothers who delivered during the study period (Figure 1), 834 (5.4%) had thrombocytopenia. Of all mothers, 6487 met the inclusion criteria; 485 (7.5%) were diagnosed with mild thrombocytopenia, whereas 6002 had a normal platelet count.

Table 1 summarizes the baseline characteristics of the pregnant women included in this study. Among mothers who experienced PPH, the mild thrombocytopenia group had significantly higher rates of uterine atony and intensive care unit admission compared with the normal platelet count group, but the mean total hospital stay did not differ between the two groups.

**Figure 2:**
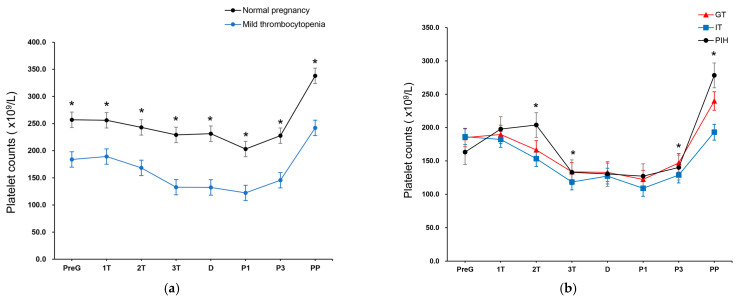
Mean platelet count over time. Mean platelet counts with 95% confidence interval at PreG state, three trimesters of antenatal period (1T, 2T, 3T), admission period (admission day [D], postoperative days 1 and 3 [P1 and P3]), and PP after 8 weeks of delivery are shown. (**a**) Trajectory of the mean platelet counts of women who had normal platelet counts and mild thrombocytopenia. * *p* < 0.015. (**b**) Trajectory of the mean platelet counts of the women with GT, IT, and PIH. * *p* < 0.015. GT, gestational thrombocytopenia; IT, immune-related thrombocytopenia; PIH, pregnancy-induced hypertension; PreG, pregestational; PP, postpartum period.

The mean platelet counts are shown in Figure 2. The mean platelet count at all time points was significantly lower in the mild thrombocytopenia group than in the normal platelet count group (Figure 2a). The mean platelet count in the mild thrombocytopenia group decreased beyond the cutoff for thrombocytopenia (150 × 10^9^/L) from the third trimester to postoperative day 3 and normalized within 8 weeks of delivery. Figure 2b shows the mean platelet count in the subgroup analysis of mild thrombocytopenia. All three subgroups showed mild thrombocytopenia from the third trimester to postoperative day 3, which recovered to normal platelet counts during the postpartum period. The mean platelet counts of women with IT were significantly lower than those of women in the other subgroups in the second and third trimesters, postoperative day 3, and postpartum period (*p* < 0.015 for all comparisons).

Table 2 presents the primary and secondary outcomes of this study. The incidence of PPH was significantly more common in women with mild thrombocytopenia than in those with a normal platelet count (1% vs. 0.3%, *p* < 0.020). Among the secondary outcomes, blood transfusion and a ≥4 g/dL drop in Hb level were also more common in women with mild thrombocytopenia. Furthermore, the association between mild thrombocytopenia and PPH was evaluated with and without adjustments for confounders. Multivariate logistic regression revealed that women with mild thrombocytopenia had increased odds of PPH (adjusted OR: 3.74; 95% CI: 1.36–10.30), blood transfusion (adjusted OR: 2.28; 95% CI: 1.57–3.33), and ≥4 g/dL drop in Hb level (2.99; 95% CI 1.86–4.82), compared with women with normal platelet counts.

The primary and secondary outcomes of GT are presented in Table 3. After excluding women with mild thrombocytopenia of other etiologies, the outcomes were similar between women with normal platelet counts and those with mild thrombocytopenia, except for PPH. The subgroup analysis revealed no significant differences in the primary and secondary outcomes of PPH among the three subgroups (Table S1).

## 4. Discussion

### 4.1. Main Findings

Among women with singleton pregnancies who underwent cesarean section, mild thrombocytopenia was independently associated with PPH. Specifically, patients with mild thrombocytopenia had a threefold higher risk of PPH than those with normal platelet counts. These women were also more likely to require blood transfusions and experience a ≥4 g/dL drop in Hb level. In the subgroup analysis, the primary and secondary outcomes of PPH among the three subgroups did not differ significantly, although the mean platelet counts of patients with IT were lower than those of the other subgroups.

### 4.2. Interpretation

Our findings demonstrating an association between mild thrombocytopenia and PPH align with previous research on women who delivered via cesarean section or vaginal birth [18,19,20,21,22]. This relationship is clinically significant, as it may influence preoperative risk assessment and management strategies for women undergoing cesarean delivery. Govindappagari et al. conducted a study on 2845 nulliparous women with term, singleton, and vertex deliveries and found that women with mild thrombocytopenia had approximately twice the likelihood of experiencing PPH compared to those with normal platelet counts [22]. This association was particularly pronounced when PPH was identified using ICD-10 codes when uterotonic agents were administered, when total blood loss reached or exceeded 1000 mL, and when blood transfusion was required. Similarly, a larger study involving 54,597 women who underwent cesarean section or vaginal birth after cesarean section also established a correlation between mild thrombocytopenia and PPH as diagnosed through laboratory and clinical evidence [20]. Furthermore, in an analysis of 1577 women who underwent elective cesarean section at term, preoperative mild thrombocytopenia was associated with an elevated risk of PPH, including an increased need for blood transfusion and a decrease in Hb levels [21].

Despite evidence supporting this association, it is important to acknowledge that some studies have reported conflicting results. Several investigations have failed to demonstrate an increased bleeding risk in mothers with mild to moderate thrombocytopenia [23,24]. For example, DiSciullo et al. compared 298 women with mild thrombocytopenia to 3133 women with normal platelet counts and found no significant differences in PPH (defined as quantitative blood loss >1000 mL), transfusion requirements, wound complications, or postpartum emergency department visits among women undergoing cesarean section [25]. These contradictory findings raise important questions regarding the consistency of this association across different populations and clinical contexts. Such discrepancies may be attributed to insufficient statistical power in some studies, particularly those with smaller sample sizes, which may not adequately capture the relationship between mild thrombocytopenia and the relatively uncommon occurrence of PPH.

When interpreting the existing literature, it is essential to recognize the inconsistency in PPH definitions and methodological criteria across studies. Many studies have examined pregnancy complications involving placental abnormalities and multiple pregnancies, which are known confounding factors when assessing the relationship between mild thrombocytopenia and PPH. Placental abnormalities, including placenta previa, placental abruption, and placenta accreta spectrum, are well-established risk factors for massive PPH [26]. Multivariable analyses of risk factors for PPH have demonstrated that multiple pregnancies carry a higher odds ratio for PPH than mild thrombocytopenia [18,21]. Additionally, a population-based study of 307,415 women revealed that emergency and elective cesarean deliveries were associated with more than threefold and twofold higher risks of PPH, respectively, compared to vaginal birth [9]. To address these methodological limitations and mitigate potential confounding factors, our study specifically examined the association between mild thrombocytopenia and PPH risk in women with singleton pregnancies undergoing cesarean delivery while excluding cases involving placental abnormalities. This methodological approach strengthens the validity of our findings by controlling for key variables known to influence PPH risk independently of thrombocytopenia status.

Another significant finding of our study, which extends beyond the existing literature, was that PPH risk did not differ significantly among subgroups with various etiologies of mild thrombocytopenia. This observation is clinically relevant, as it suggests that the underlying cause of thrombocytopenia may be less important than the presence of thrombocytopenia itself when assessing PPH risk. Previous comparative analyses of different thrombocytopenia etiologies have shown mixed results. Wang et al. categorized 195 women with thrombocytopenia into pregnancy-associated thrombocytopenia, IT, and hypertensive disorder in pregnancy groups, demonstrating similar incidences of PPH across these classifications [27]. Conversely, another study indicated that women with IT were more likely to experience moderate-to-severe thrombocytopenia and require more transfusions than other groups [28]. This discrepancy may be attributed to differences in the proportion of women with moderate-to-severe thrombocytopenia included in each study.

To the best of our knowledge, this study is the first comprehensive evaluation of the association between various causes of thrombocytopenia, particularly mild thrombocytopenia, and PPH. This contribution to the literature is particularly valuable for clinicians managing pregnant women with mild preoperative thrombocytopenia of diverse origins. A definitive diagnosis is often not possible until after delivery, as GT and IT cannot be distinguished based solely on laboratory tests. Based on our findings, preoperative mild thrombocytopenia appears to confer a higher risk of PPH compared to normal platelet counts but demonstrates a similar prognosis regardless of the underlying cause. These findings have important implications for clinical practice, as obstetricians should consider mild thrombocytopenia a risk factor for PPH when planning cesarean deliveries, regardless of the suspected cause of low platelet count.

### 4.3. Strengths and Limitations

The main limitation of this study was its retrospective design. Although we aimed to control for all relevant confounders, residual confounding factors may still be present. Additionally, we did not evaluate treatments for thrombocytopenia other than transfusion or the timing of platelet transfusion (preoperative vs. postoperative). Furthermore, the use of ICD-10 codes for diagnosing PPH may have introduced subjectivity; therefore, we supplemented our analysis with additional clinical and laboratory data.

Despite these limitations, this study has several strengths. To the best of our knowledge, this is the first study to investigate the effect of mild thrombocytopenia on PPH across different diagnoses and to examine the longitudinal trend of laboratory results from the pregestational state to 8 weeks postpartum. A major strength of this study is its large cohort size. Our cohort included only women who underwent cesarean deliveries in tertiary care centers, which serve as referral hospitals for pregnant women with complications, particularly hematological disorders, enhancing the reliability of our findings. By excluding women with multiple pregnancies and placental diseases, we minimized the inclusion of patients with severe morbidities that could have biased our results.

## 5. Conclusions

Overall, we found that women with mild thrombocytopenia who underwent cesarean section had a higher risk of developing PPH and required more blood transfusions. Therefore, clinicians should remain vigilant when managing patients with mild thrombocytopenia on the day of delivery, even when the underlying cause is unclear. Large-scale prospective studies are necessary to further investigate the impact of mild thrombocytopenia on maternal health and to refine management strategies for pregnant women with this condition.

## Figures and Tables

**Table 1:** Baseline characteristics of the pregnant women in the study.

	Normal Platelet Counts (*n* = 6002)	Mild Thrombocytopenia (*n* = 485)	*p-*Value
Maternal age, years *	33.7 ± 4.1	33.8 ± 4.0	0.549
Primiparity, *n* (%)	3278 (54.6)	278 (57.3)	0.270
Gestational age at delivery, wk	36.9 ± 3.1	37.0 ± 2.9	0.701
Neonatal birth weight, kg	2.9 ± 0.7	2.9 ± 0.8	0.304
Pregestational BMI (kg/m^2^)	21.0 ± 3.7	20.6 ± 4.0	0.057
Pregestational AA	5 (0.1)	4 (0.8)	<0.001
Pregestational ITP	19 (0.3)	4 (0.8)	0.157
Pregestational hypertension, *n* (%)	302 (5.0)	15 (3.1)	0.073
Pregestational SLE	51 (0.8)	15 (3.1)	<0.001
Pregestational APS syndrome	21 (0.3)	11 (2.3)	<0.001
Previous uterine operation	890 (14.8)	50 (10.3)	0.008
Emergency cesarean section, *n* (%)	2937 (48.9)	256 (52.9)	0.103
Pregnancy-induced hypertension, *n* (%)	455 (7.6)	67 (13.8)	<0.001
Gestational diabetes mellitus, *n* (%)	371 (6.2)	29 (6.0)	0.936
Uterine atony, *n* (%)	5 (0.1)	2 (0.4)	0.034
Hospital days, d *	4.3 ± 5.1	4.2 ± 3.4	0.382

Values are expressed as mean ± standard deviation or * number (*n*, %). BMI, body mass index; AA, aplastic anemia; ITP, immune thrombocytopenic purpura; SLE, systemic lupus erythematosus; APS, antiphospholipid syndrome.

**Table 2:** Primary and secondary outcomes in the study.

	Normal Platelet Counts (*n* = 6002)	Mild Thrombocytopenia (*n* = 485)	*p*-Value	Crude OR (95% CI)	aOR (95% CI)
Postpartum hemorrhage, *n* (%)	17 (0.3)	5 (1.0)	0.020	3.67 (1.35, 9.98)	3.74 (1.36, 10.30)
Intrauterine device, *n* (%)	68 (1.1)	9 (1.9)	0.232	1.65 (0.82, 3.33)	1.02 (0.99, 1.04)
Uterine artery embolization, *n* (%)	3 (0.0)	1 (0.2)	0.183	4.13 (0.43, 39.79)	3.64 (0.38, 35.29)
Hysterectomy, *n* (%)	5 (0.1)	0 (0)	0.524	0	0
Blood transfusion, *n* (%)	194 (3.2)	35 (7.2)	<0.001	2.33 (1.60, 3.38)	2.28 (1.57, 3.33)
≥4 g/dL drop in hemoglobin level	92 (1.5)	22 (4.5)	<0.001	3.05 (1.90, 4.90)	2.99 (1.86, 4.82)

Values are expressed as mean ± standard error or number (n, %). aOR was determined by multivariate logistic regression adjusted for maternal age, gestational age at delivery, body mass index, and previous abdominal surgery, including cesarean delivery. OR, odds ratio; aOR, adjusted odds ratio; CI, confidence interval.

**Table 3:** Primary and secondary outcomes of GT after excluding women with IT and PIH.

	Normal Platelet Counts (*n* = 5487)	Mild Thrombocytopenia (*n* = 400)	*p*-Value	Crude OR (95% CI)	aOR (95% CI)
Postpartum hemorrhage, *n* (%)	13 (0.2)	2 (0.5)	0.314	2.12 (0.48, 9.41) *p* = 0.325	2.14 (0.48, 9.55) *p* = 0.321
Intrauterine device, *n* (%)	66 (1.2)	8 (2)	0.167	1.68 (0.80 3.52) *p* = 0.172	1.70 (0.81, 3.58) *p* = 0.164
Uterine artery embolization, *n* (%)	3 (0.1)	1 (0.2)	0.148	4.58 (0.48, 44.15) *p* = 0.188	4.72 (0.47, 47.00) *p* = 0.186
Hysterectomy, *n* (%)	5 (0.1)	0 (0)	0.546	0	0
Blood transfusion, *n* (%)	168 (3.1)	20 (5)	0.033	1.67 (1.04, 2.68) *p* = 0.035	1.73 (1.07, 2.79) *p* = 0.025
≥4 g/dL drop in hemoglobin level	76 (1.4)	16 (4)	<0.001	2.97 (1.71, 5.13) *p* < 0.001	2.88 (1.66, 5.00) *p* < 0.001

Values are expressed as mean ± standard error or number (n, %). aOR was determined by multivariate logistic regression adjusted for maternal age, gestational age at delivery, body mass index, and previous abdominal surgery, including cesarean delivery. GT, gestational thrombocytopenia; IT, immune-mediated thrombocytopenia; PIH, pregnancy-induced hypertension; OR, odds ratio; aOR, adjusted odds ratio; CI, confidence interval.

## Data Availability

Data are contained within the article.

## Supplementary Materials

The following supporting information can be downloaded at: https://www.mdpi.com/article/10.3390/jcm14062031/s1, Table S1: Primary and secondary outcomes within subgroups.

## Abbreviations

AA	aplastic anemia
aOR	adjusted odds ratio
APS	antiphospholipid syndrome
BMI	body mass index
CI	confidence interval
GT	gestational thrombocytopenia
Hb	hemoglobin
HELLP	hemolysis, elevated liver enzymes, low platelet count
ICU	intensive care unit
IT	immune-mediated thrombocytopenia
ITP	immune thrombocytopenic purpura
OR	odds ratio
PIH	pregnancy-induced hypertension
PP	postpartum period
PPH	postpartum hemorrhage
PreG	pregestational
RBC	red blood cell
SLE	systemic lupus erythematosus
UAE	uterine artery embolization
