# Supplementary material for: Association Between Mild Thrombocytopenia Prior to Cesarean Section and Postpartum Hemorrhage
Source: J Clin Med. 2025 Mar 17;14(6):2031. doi: 10.3390/jcm14062031 (PMC11943230; doi:10.3390/jcm14062031)
Supplement: Supplementary file 1 [file jcm-14-02031-s001.zip › jcm-3472995-supplementary.pdf]

Table S1. Primary and secondary outcomes within subgroups

	GT (n=408)	IT (n=21)	PIH (n=56)	*p*-value	aOR (95% CI)*	
					IT	PIH
Postpartum hemorrhage	2 (0.5)	0 (0)	3 (5.4)	0.003	0	7.83 (0.77 79.53)
Intrauterine device	8 (2.0)	0 (0)	1 (1.8)	0.809	0	0.82 (0.07 9.31)
Uterine artery embolization	1 (0.2)	0 (0)	0 (0)	0.910	0	0
Blood transfusion	21 (5.1)	4 (19.0)	10 (17.9)	<0.001	3.43 (0.97 12.06)	2.44 (0.91 6.58)
Hemoglobin drop ≥ 4g/dL	17 (4.2)	0 (0)	5 (8.9)	0.164	0	2.12 (0.61 7.35)

The values are expressed as number (%).

aOR was determined by multivariate logistic regression adjusted for maternal age, gestational age at delivery, body mass index, and previous abdominal surgery including cesarean delivery.

Abbreviations: GT, gestational thrombocytopenia; IT, immune-related thrombocytopenia; PIH, pregnancy-induced hypertension; aOR, adjusted odds ratio; CI, confidence interval.

* aOR for IT and PIH were calculated with GT as the reference.
